# Postoperative Delirium in the Oldest–Old: Parallel Analyses of Institutional and National Surgical Cohorts

**DOI:** 10.1002/brb3.71332

**Published:** 2026-04-24

**Authors:** Shan Hung, Chih‐Yau Chang, Kuo‐Hsuan Chung

**Affiliations:** ^1^ Department of Psychiatry Taipei Medical University Hospital Taipei Taiwan; ^2^ Psychiatric Research Center Taipei Medical University Hospital Taipei Taiwan; ^3^ Quality Management Department Taipei Medical University Hospital Taipei Taiwan; ^4^ Department of Psychiatry, School of Medicine, College of Medicine Taipei Medical University Taipei Taiwan

**Keywords:** ACS‐NSQIP, ASA classification, delirium screening, geriatric surgery, oldest–old, postoperative delirium, serum albumin, white blood cell count

## Abstract

**Background:**

Postoperative delirium (POD) is a common and serious complication in elderly surgical patients, particularly among the oldest–old (aged ≥ 75 years). Evidence regarding clinical factors associated with POD in this population remains limited, especially across different healthcare settings. This study examined patterns of clinical characteristics associated with POD in two surgical cohorts analyzed in parallel: a national US surgical database and a single‐center Taiwanese cohort.

**Methods:**

We conducted a retrospective cohort study using data from the American College of Surgeons National Surgical Quality Improvement Program (ACS‐NSQIP) and Taipei Medical University Hospital (TMUH). Patients aged ≥ 75 years who underwent surgery under general anesthesia and entered a POD assessment process as part of routine clinical care were included. Preoperative clinical variables and laboratory parameters were analyzed. Univariate and multivariable logistic regression analyses were performed separately for each cohort to examine associations with POD among clinically evaluated patients.

**Results:**

A total of 2598 patients were included in the analytic population (ACS‐NSQIP: *n* = 1316; TMUH: *n* = 1282), among whom 212 (8.16%) were identified with POD. The proportion of POD identified on screening was higher in the ACS‐NSQIP cohort (11.17%) than in the TMUH cohort (5.07%). In the ACS‐NSQIP cohort, higher American Society of Anesthesiologists (ASA) physical status, abnormal preoperative serum albumin, and abnormal white blood cell count were independently associated with POD. In the TMUH cohort, older age and higher ASA class were significantly associated with POD. Associations involving serum albumin in the TMUH cohort should be interpreted cautiously due to substantial missingness, which likely reflects nonrandom laboratory testing practices rather than biological effects. Differences in patient characteristics between cohorts included age, body mass index, operative time, and laboratory profiles.

**Conclusion:**

Among oldest–old surgical patients who underwent POD assessment in routine clinical practice, patterns of clinical factors associated with POD differed across the two cohorts analyzed. These findings should be interpreted in the context of differences in screening practices, case‐mix, and data completeness, and do not represent population‐based incidence estimates. Prospective studies incorporating standardized delirium screening protocols are needed to support the development of robust and generalizable risk stratification approaches.

## Graphical abstract

This study compared factors associated with postoperative delirium (POD) among the oldest–old surgical patients (aged ≥ 75 years) in a national US cohort (ACS‐NSQIP) and a single‐center Taiwanese cohort (TMUH). Among clinically evaluated patients, POD was associated with higher ASA physical status, abnormal serum albumin levels, and abnormal white blood cell counts in the ACS‐NSQIP cohort, whereas older age and higher ASA class were associated with POD in the TMUH cohort. Differences in albumin‐related findings likely reflect cohort‐specific clinical assessment and laboratory testing practices.

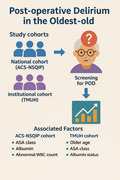

## 1 Introduction

A growing number of older adults are undergoing anesthesia and surgery to improve functional abilities and quality of life through the treatment of various diseases. In Western countries, approximately 37% of all surgeries are performed on individuals over the age of 65 (Centers for Disease Control and Prevention 2010). According to recent analyses, postoperative delirium (POD) occurs in 18%–30% of older patients undergoing noncardiac surgery, with incidence varying by surgery type, for example, up to 32% in cardiac surgery and 20% in orthopedic procedures (Igwe et al. 2023; Sadeghirad et al. 2023; Suzuki et al. 2025). Moreover, about 10%–40% of these patients may experience persistent cognitive impairment or functional decline within months to a year following surgery (Sadeghirad et al. 2023).

Defined as an “acute confusional state,” delirium is characterized by disturbances in attention and cognition, a fluctuating course of symptoms, and manifestations that cannot be attributed to preexisting cognitive disorders. Clinical evidence suggests that these disturbances may arise from various factors, including medical conditions, substance intoxication or withdrawal, and medication side effects (APA 2013). The clinical presentation of delirium includes several subtypes based on psychomotor activity: hyperactive, hypoactive, mixed, and unclassified (Liptzin and Levkoff 1992; O'Keeffe and Lavan 1999). The hyperactive variant, characterized by increased psychomotor activity, is the most easily recognized. Patients with hyperactive delirium may exhibit agitation, psychosis, mood lability, and noncooperative behaviors, sometimes resulting in injuries from falls, combativeness, or the removal of medical devices. Conversely, hypoactive delirium presents as sluggishness, lethargy, or apparent low mood with underlying confusion that isn't immediately evident. Most patients experience a mixture of both hypoactive and hyperactive delirium due to multiple etiological factors and fluctuating symptom courses. Studies suggest that mixed‐type delirium presents the highest risk for substantial morbidity and mortality (O'Keeffe and Lavan 1999).

Delirium has multiple adverse consequences, leading to high morbidity in hospitalized patients due to increased risks of dehydration, malnutrition, falls, continence issues, and pressure sores. Patients with delirium face elevated 1‐year mortality rates (35%–40%), higher readmission rates, and an increased likelihood of institutionalization (47% vs. 18%) (George et al. 1997; Inouye et al. 1998). Delirium also contributes to prolonged hospitalization and elevated healthcare costs (McCusker et al. 2003; Rizzo et al. 2001). While a 2004 Medicare analysis estimated an additional cost of $2500 per patient—totaling $6.9 billion annually—this figure likely underrepresents the current burden due to inflation and increased surgical volume among older adults (Inouye et al. 2001).

Although delirium poses potentially life‐threatening risks, early recognition and preventative strategies can effectively reduce its incidence. Various predictors have been consistently identified in recent studies, including advanced age, male sex, preoperative cognitive impairment or dementia, hypoalbuminemia, multiple comorbidities, and prolonged operative time (Sadeghirad et al. 2023; Suzuki et al. 2025). Analyses from the American College of Surgeons National Surgical Quality Improvement Program (ACS‐NSQIP) have further identified additional risk factors among patients undergoing open reduction and internal fixation (ORIF) for hip fractures, such as age ≥ 65 years, partially dependent functional status, bleeding disorders, preoperative delirium or dementia, nonemergency admission, and American Society of Anesthesiologists (ASA) physical status classification greater than II (Maliket al. 2019), which alone has been associated with a more than twofold increase in delirium risk (Sadeghirad et al. 2023).

Taiwan entered an “aging society” (defined as 14% of the population aged 65 or older) at the end of 2016. At the end of 2023, the total population was 23,424,422, with 4,296,985 individuals aged 65 or older (18.35% of the total population). National Development Council projections indicate Taiwan will become a “super‐aged society” (defined as over 20% of the population aged 65 or older) in 2025, with this proportion rising to 43.6% by 2070 (Ministry of the Interior Taiwan 2024). While the challenges to healthcare quality posed by an aging population are becoming increasingly prominent, Taiwanese research on POD in the elderly is underdeveloped. This study focuses on the “oldest–old,” generally defined as individuals aged 75 years and older, who are especially susceptible to postoperative complications such as delirium. In this study, we examined clinical characteristics associated with POD among oldest–old surgical patients who underwent POD assessment as part of routine clinical care. Using data from a national US surgical database (ACS‐NSQIP) and a single‐center cohort from northern Taiwan, we conducted parallel analyses to explore patterns of association within distinct clinical contexts. Rather than estimating population‐based incidence, our objective was to characterize associations observed in clinically evaluated cohorts, with the aim of informing context‐specific risk stratification and future development of tailored preventive strategies for POD.

## 2 Methods

### 2.1 Database

ACS‐NSQIP is a nationally validated, risk‐adjusted, outcomes‐based program to measure and improve the quality of surgical care. The dataset includes clinical information collected prospectively from participating hospitals across the United States (ACS 2021). Data are abstracted by trained surgical clinical reviewers, with audit reports indicating an inter‐reviewer disagreement rate of less than 2% (Shiloach et al. 2010).

Taipei Medical University Hospital (TMUH) is a regional teaching hospital in Northern Taiwan with a capacity of over 800 beds.

We analyzed data from the 2021 ACS‐NSQIP public use file and from TMUH between October 2020 and April 2023. Clinical characteristics and perioperative variables were retrospectively extracted from the medical records of surgical patients.

### 2.2 Inclusion and Exclusion Criteria

A total of 66,667 surgical patients were initially screened. Following cohort‐specific restriction steps to patients aged ≥ 75 years who underwent surgery under general anesthesia, patients who did not enter a POD assessment workflow as part of routine clinical care were excluded. Patients with missing core demographic variables required for cohort definition (e.g., age or sex) were excluded prior to analysis.

**FIGURE 1:**
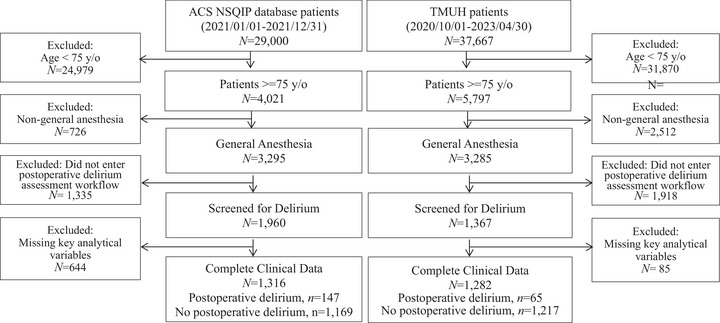
Patient selection and analytic cohort derivation. The flow diagram illustrates the stepwise selection of older surgical patients from the ACS‐NSQIP and Taipei Medical University Hospital (TMUH) databases. Exclusions were applied sequentially based on age (< 75 years), absence of general anesthesia, nonentry into a postoperative delirium assessment workflow, and missing key analytical variables. The final analytic population comprised patients who underwent postoperative delirium assessment as part of routine clinical practice. Abbreviations: ACS‐NSQIP, American College of Surgeons National Surgical Quality Improvement Program; TMUH, Taipei Medical University Hospital.

In the TMUH cohort, laboratory testing was performed as part of routine clinical practice rather than within a standardized registry protocol. Therefore, laboratory variables exhibited substantial missingness. To preserve sample size and reflect real‐world clinical decision‐making, missing laboratory values were retained and modeled as a separate category in regression analyses. Non‐laboratory variables had minimal missingness (e.g., BMI, 0.6%). Analyses were conducted using available data. After applying these criteria, 2598 patients were included in the analytic population, comprising 212 patients identified with POD (POD group) and 2386 patients without POD (non‐POD group), drawn from both the ACS‐NSQIP and TMUH datasets (Figure 1).

### 2.3 Assessment Tools

At TMUH, delirium assessment was performed using a modified confusion assessment method (CAM) as part of routine clinical care. This assessment evaluated acute onset or fluctuating course, inattention, disorganized thinking, and altered level of consciousness. Delirium assessments were conducted based on clinical judgment rather than universal screening.

In the ACS‐NSQIP database, delirium was recorded as “delirium present on screening” and could be assessed using various validated instruments, including the CAM, CAM‐ICU, brief CAM, Delirium Rating Scale, DRS‐R‐98, Memorial Delirium Assessment Scale, Nursing Delirium Screening Checklist, Single Question in Delirium, and the 4As Test.

### 2.4 Baseline Demographic, Clinical, and Laboratory Variables

Demographic variables included age, sex, and body mass index (BMI). Perioperative clinical characteristics included ASA physical status classification, operative time, and length of hospital stay.

Preoperative laboratory variables were categorized as normal or abnormal using predefined clinical thresholds. Abnormal values were defined as follows: potassium < 3.0 or > 5.5 mmol/L; estimated glomerular filtration rate (eGFR) < 30 mL/min/1.73 m^2^; C‐reactive protein (CRP) > 9 mg/L; serum albumin < 3.5 g/dL; white blood cell (WBC) count < 3000 or > 10,000 cells/µL; hemoglobin < 12.0 g/dL; and platelet count < 100,000 cells/µL. Laboratory variables were dichotomized for inclusion in regression analyses.

In the ACS‐NSQIP cohort, all laboratory data were complete. In contrast, substantial missingness was observed in the TMUH cohort, particularly for serum albumin. To preserve sample size and reflect routine clinical testing practices, missing laboratory values in the TMUH cohort were retained and analyzed as a separate category in regression analyses.

### 2.5 Statistical Analysis

Patients were classified as POD or non‐POD. Continuous variables were summarized as means ± standard deviations and compared using independent‐samples *t*‐tests. Categorical variables were compared using chi‐square or Fisher's exact tests, as appropriate.

Multivariable logistic regression models were constructed separately for the ACS‐NSQIP and TMUH cohorts to explore associations between clinical variables and POD among clinically evaluated patients. Variables demonstrating statistically significant associations in univariate analyses were considered for inclusion to construct parsimonious multivariable models, given the limited number of POD events in the TMUH cohort. Adjusted odds ratios (aORs) with 95% confidence intervals (CIs) were reported.

All analyses were performed using IBM SPSS Statistics version 28.0 (IBM Corp., Armonk, New York, USA). Two‐tailed *p*‐values < 0.05 were considered statistically significant.

## 3 Results

Patients aged ≥ 75 years who underwent general anesthesia were identified from the ACS‐NSQIP (*n* = 3295) and TMUH (*n* = 3285) datasets. After excluding patients who did not enter the POD assessment workflow or who lacked essential demographic variables required for analysis, a total of 2598 patients were included in the analytic population (ACS‐NSQIP: *n* = 1316; TMUH: *n* = 1282), including 212 patients diagnosed with POD (ACS‐NSQIP: *n* = 147; TMUH: *n* = 65).

The proportion of patients with POD on screening was 11.17% in the ACS‐NSQIP cohort (147/1316) and 5.07% in the TMUH cohort (65/1282), as shown in Table 1.

**TABLE 1:** Prevalence of postoperative delirium among patients included in the analytic population of the ACS‐NSQIP and TMUH cohorts.

Dataset	Total patients (*n*)	POD cases (*n*)	Prevalence (%)
ACS‐NSQIP	1316	147	11.17
TMUH	1282	65	5.07

*Note*: The proportion of patients with POD among those who underwent postoperative delirium assessment and were included in the analytic population.

Abbreviations: ACS‐NSQIP, American College of Surgeons National Surgical Quality Improvement Program; *n*, number; POD, postoperative delirium; TMUH, Taipei Medical University Hospital.

### 3.1 ACS‐NSQIP Cohort

Table 2 presents the comparison of baseline characteristics between the POD and non‐POD groups in the ACS‐NSQIP dataset. Significant associations with POD were observed for a history of chronic obstructive pulmonary disease (COPD) (*p* = 0.016), congestive heart failure (CHF) (*p* = 0.005), dialysis (*p* = 0.038), abnormal eGFR (*p* = 0.002), low preoperative serum albumin levels (*p* < 0.001), abnormal WBC count (*p* < 0.001), and higher ASA physical status classification (*p* < 0.001).

**TABLE 2:** Baseline characteristics of patients with and without postoperative delirium in the ACS‐NSQIP cohort.

		POD (*n* = 147)		Non‐POD (*n* = 1169)		*p* value
Categorical variables		*n*	%	*n*	%	
Gender						0.327
	Female	85	12.0	626	88.0	
	Male	62	10.2	543	89.8	
Age						0.098^a^
	≧ 90	0	0	0	0	
	85 ∼ 89	28	14.1	170	85.9	
	80 ∼ 84	48	11.7	363	88.3	
	75 ∼ 79	71	10.0	636	90.0	
Smoking						0.317
	No	137	11.0	1112	89.0	
	Yes	10	14.9	57	85.1	
**Medical history**						
Diabetes						0.118
	No	110	10.5	939	89.5	
	Yes	37	13.9	230	86.1	
COPD						0.016*
	No	130	10.6	1096	89.4	
	Yes	17	18.9	73	81.1	
CHF						0.005**
	No	134	10.7	1124	89.3	
	Yes	13	22.4	45	77.6	
Dialysis						0.038*
	No	143	11.0	1159	89.0	
	Yes	4	28.6	10	71.4	
Cancer						0.098
	No	133	10.8	1099	89.2	
	Yes	14	16.7	70	83.3	
**Preoperative laboratory values**						
eGFR						0.002**
	Normal	133	10.6	1124	89.4	
	Abnormal	14	23.7	45	76.3	
Serum albumin						< 0.001**
	Normal	92	8.9	937	91.1	
	Abnormal	55	19.2	232	80.8	
WBC count						< 0.001**
	Normal	100	9.5	951	90.5	
	Abnormal	47	17.7	218	82.3	
Platelet						0.083
	Normal	142	11.0	1152	89.0	
	Abnormal	5	22.7	17	77.3	
ASA class						< 0.001**^a^
	I	0	0	0	0	
	II	14	6.2	212	93.8	
	III	96	10.3	834	89.7	
	IV	35	22.4	121	77.6	
	V	2	50.0	2	50.0	
Continuous variables		M	SD	M	SD	
Age		80.4	4.0	79.8	3.8	0.062
BMI		26.0	8.7	26.6	6.7	0.423
Operating time (minutes)		193.3	127.8	184.7	116.7	0.407
eGFR		68.0	30.6	72.2	26.7	0.113
Serum albumin		3.6	0.8	3.9	0.6	< 0.001**
WBC count		8.9	5.1	8.0	3.8	0.033*
Platelet		238.0	100.1	245.9	92.5	0.335

*Note*: Abnormal laboratory values were defined according to predefined clinical thresholds as described in Methods. No missing data were present for the variables included in this analysis. *p* values were calculated using chi‐square test for categorical variables and independent *t*‐test for continuous variables unless otherwise specified.

Abbreviations: ASA, American Society of Anesthesiologists physical status classification system; ACS‐NSQIP, American College of Surgeons National Surgical Quality Improvement Program; BMI, body mass index; CHF, congestive heart failure; COPD, chronic obstructive pulmonary disease; eGFR, estimated glomerular filtration rate; M, mean; *n*, number; POD, postoperative delirium; SD, standard deviation; WBC, white blood cell (count).

^a^ Linear‐by‐linear association test.

*p < 0.05; **p < 0.01.

### 3.2 TMUH Cohort

As shown in Table 3, POD was significantly associated with older age (*p* = 0.003), a history of COPD (*p* = 0.015), abnormal eGFR (*p* = 0.014), low preoperative serum albumin levels (*p* < 0.001), abnormal WBC count (*p* = 0.016), and higher ASA classification (*p* < 0.001). Other factors, including smoking status, diabetes, heart failure, dialysis, cancer, platelet count, BMI, and operative time, were not significantly different between the POD and non‐POD groups.

**TABLE 3:** Baseline characteristics of patients with and without postoperative delirium in the TMUH cohort.

		POD (*n* = 65)		Non‐POD (*n* = 1217)		*p* value
Categorical variables		*n*	%	*n*	%	
Gender						0.264
	Female	30	4.4	648	95.6	
	Male	35	5.8	569	94.2	
Age						0.003**^a^
	≧ 90	13	13.7	82	86.3	
	85 ∼ 89	11	5.3	195	94.7	
	80 ∼ 84	17	4.2	389	95.8	
	75 ∼ 79	24	4.2	551	95.8	
Smoking						0.545^b^
	No	61	5.0	1160	95.0	
	Yes	4	6.6	57	93.4	
**Medical history**						
Diabetes						0.113
	No	43	4.5	912	95.5	
	Yes	22	6.7	305	93.3	
COPD						0.015*^b^
	No	59	4.8	1182	95.2	
	Yes	6	14.6	35	85.4	
CHF						0.306^b^
	No	59	4.9	1140	95.1	
	Yes	6	7.2	77	92.8	
Dialysis						0.737^b^
	No	62	5.0	1170	95.0	
	Yes	3	6.0	47	94.0	
Cancer						0.150
	No	63	5.3	1120	94.7	
	Yes	2	2.0	97	98.0	
**Preoperative laboratory values**						
eGFR						0.014*
	Normal	37	4.0	878	96.0	
	Abnormal	10	10.0	90	90.0	
	Missing	18	6.7	249	93.3	
Serum Albumin						< 0.001**
	Normal	13	11.2	103	88.8	
	Abnormal	12	13.2	79	86.8	
	Missing	40	3.7	1035	96.3	
WBC count						0.016*
	Normal	31	3.8	793	96.2	
	Abnormal	11	7.2	141	92.8	
	Missing	23	7.5	283	92.5	
Platelet						0.090
	Normal	43	4.5	910	95.5	
	Abnormal	0	0	24	100.0	
	Missing	22	7.2	283	92.8	
ASA class						< 0.001**^a^
	I	0	0	1	100	
	II	10	2.0	489	98.0	
	III	30	4.5	630	95.5	
	IV	25	20.5	97	79.5	
	V	0	0	0	0	
Continuous variables		M	SD	M	SD	
Age		84.2	7.4	81.6	4.9	0.008**
BMI		23.8	4.0	23.9	3.9	0.753
Operating time(minutes)		130.3	138.7	121.1	106.2	0.503
eGFR		63.8	41.5	67.4	28.5	0.562
Serum albumin		3.5	0.7	3.4	1.0	0.742
WBC count		8.4	4.3	7.6	3.4	0.237
Platelet		203.5	85.5	220.4	81.5	0.185

*Note*: BMI was missing in eight patients (0.6%). Missing laboratory values were retained as a separate category in multivariable analyses to preserve sample size. Analyses were based on available data. *p* values were calculated using chi‐square test for categorical variables and independent *t*‐test for continuous variables unless otherwise specified.

Abbreviations: ASA, American Society of Anesthesiologists physical status classification system; BMI, body mass index; CHF, congestive heart failure; COPD, chronic obstructive pulmonary disease; eGFR, estimated glomerular filtration rate; M, mean; *n*, number; POD, postoperative delirium; SD, standard deviation; TMUH, Taipei Medical University Hospital; WBC, white blood cell (count).

^a^ Linear‐by‐ linear association test.

^b^ Fisher's exact test.

*p < 0.05; **p < 0.01.

### 3.3 Multivariable Logistic Regression Analyses

In the ACS‐NSQIP cohort (Table 4A), higher ASA physical status classification was significantly associated with increased risk of POD (aOR 1.72; 95% CI, 1.23–2.40; *p* = 0.001). Additionally, patients with abnormal preoperative serum albumin had a significantly higher risk of POD (aOR 1.72; 95% CI, 1.16–2.55; *p* = 0.007), as did those with abnormal WBC count (aOR 1.71; 95% CI, 1.15–2.55; *p* = 0.008). Other variables, including age, history of COPD, CHF, dialysis, abnormal eGFR, and thrombocytopenia, were not significantly associated with POD after adjustment. In the TMUH cohort (Table 4B), older age was significantly associated with POD (aOR 1.05 per year increase; 95% CI, 1.01–1.10; *p* = 0.029). Higher ASA class was strongly associated with POD (aOR 3.14; 95% CI, 2.00–4.92; *p* < 0.001). Albumin level was overall associated with POD risk (*p* = 0.031), patients with missing albumin values showed significantly lower odds of POD compared with those with normal albumin levels, a finding that should be interpreted cautiously given nonrandom laboratory testing (aOR = 0.44; 95% CI 0.22–0.89). No significant associations were found for history of COPD, abnormal WBC count, or abnormal eGFR.

**TABLE 4A:** Multivariable logistic regression analysis for postoperative delirium in the ACS‐NSQIP cohort.

Variable	Adjusted OR (Exp(B))	95% CI	*p* value
Age (per year)	1.03	0.99–1.08	0.165
History of COPD	1.50	0.83–2.69	0.178
History of CHF	1.70	0.86–3.36	0.126
History of dialysis	1.15	0.29–4.62	0.847
Abnormal eGFR	1.50	0.70–3.22	0.303
Abnormal preoperative serum albumin	1.72	1.16–2.55	0.007**
Abnormal preoperative WBC count	1.71	1.15–2.55	0.008**
ASA class (per level)	1.72	1.23–2.40	0.001**

**p < 0.01.

**TABLE 4B:** Multivariable logistic regression analysis for postoperative delirium in the TMUH cohort.

Variable	Adjusted OR (Exp(B))	95% CI	*p* value
Age (per year)	1.05	1.01–1.10	0.029*
History of COPD	2.13	0.80–5.71	0.132
eGFR			0.645
Normal	Reference	—	—
Abnormal	1.32	0.60–2.90	0.496
Missing	0.84	0.34–2.04	0.693
Preoperative serum albumin			0.031*^a^
Normal	Reference	—	—
Abnormal	0.92	0.37–2.26	0.849
Missing	0.44	0.22–0.89	0.023*
Preoperative WBC count			0.886
Normal	Reference	—	—
Abnormal	1.02	0.47–2.23	0.952
Missing	1.24	0.53–2.90	0.628
ASA class (per level)	3.14	2.00–4.92	< 0.001**

Abbreviations: ASA, American Society of Anesthesiologists physical status classification system; CHF, congestive heart failure; CI, confidence interval; COPD, chronic obstructive pulmonary disease; eGFR, estimated glomerular filtration rate; OR, odds ratio; POD, postoperative delirium; SD, standard deviation; TMUH, Taipei Medical University Hospital; WBC, white blood cell (count).

^a^ Omnibus *p* value for the overall effect of the categorical variable (Wald *χ^2^* test across all levels).

*p < 0.05; **p < 0.01.

### 3.4 Comparison of Key Characteristics Among POD Patients in ACS‐NSQIP and TMUH Cohorts

A detailed comparison of demographic, clinical, and laboratory characteristics among POD patients in the ACS‐NSQIP and TMUH cohorts is presented in Table S1. Patients in the TMUH cohort were significantly older than those in the ACS‐NSQIP cohort (84.14 ± 7.39 vs. 80.42 ± 4.02 years, *p* < 0.001). They also had a lower BMI (23.77 ± 4.01 vs. 26.00 ± 8.74, *p*  =  0.012) and shorter operative time (130.3 ± 138.7 vs. 193.3 ± 127.8 min, *p*  =  0.002). The ACS‐NSQIP cohort exhibited a significantly higher preoperative platelet count (238.0 ± 100.1 vs. 203.5 ± 85.39, *p*  =  0.041). Categorical comparisons revealed that the TMUH cohort had a higher prevalence of abnormal eGFR (20.00% vs. 9.52%, *p*  =  0.035), whereas the ACS‐NSQIP cohort had a significantly higher proportion of patients with abnormal preoperative serum albumin (41.50% vs. 16.92%, *p* < 0.001). ASA classification also significantly differed between the two cohorts (*p*  =  0.031).

## 4 Discussion

This study examined clinical characteristics associated with POD among older surgical patients who underwent delirium assessment in routine clinical practice, using data from a national US surgical database and a single‐center Taiwanese cohort. To our knowledge, this is the first study to compare patterns of association observed in clinically evaluated cohorts from the ACS‐NSQIP and a Taiwanese hospital participating in the NSQIP program. Among patients who underwent POD assessment, the proportion of POD identified on screening was lower in the TMUH cohort (5.07%) than in the ACS‐NSQIP cohort (11.17%). This finding is consistent with a previously reported POD proportion of approximately 5.5% in a Taiwanese cohort of older patients undergoing abdominal cancer surgery (Lai et al. 2023). Similarities in patient populations and assessment practices may partially account for this consistency, although under‐recognition of hypoactive delirium in routine clinical settings cannot be excluded.

Multivariable analyses demonstrated distinct patterns of association across the two cohorts. In the ACS‐NSQIP cohort, higher ASA physical status classification was significantly associated with POD, consistent with prior literature identifying global disease burden and physiological reserve as key contributors to delirium vulnerability. Abnormal preoperative serum albumin levels and abnormal WBC counts were also independently associated with POD, supporting the roles of nutritional status and systemic inflammatory activity in delirium pathophysiology. In contrast, in the TMUH cohort, older age and higher ASA class were significantly associated with POD, while abnormal laboratory values did not retain independent associations after adjustment.

Age remains an essential consideration in understanding vulnerability to delirium. This observation aligns with existing literature that associates advanced age with a heightened risk of cognitive disturbances following surgery (Mahanna‐Gabrielli et al. 2019). Interactions between anesthetic agents and the central nervous system cholinergic pathways may be of particular importance (Praticò et al. 2005), due to the close relationship between acetylcholine and cognition. Age‐related decline in prefrontal cholinergic neurons may render the elderly more susceptible than younger patients to anesthesia‐mediated depression of CNS cholinergic neurotransmission (Fodale et al. 2006; Jansson et al. 2004). In the present study, older age was significantly associated with POD in the TMUH cohort but not in the ACS‐NSQIP cohort after adjustment. This discrepancy may reflect differences in population characteristics, healthcare delivery structures, or collinearity with other measures of physiological vulnerability, such as ASA classification and nutritional status.

The role of nutritional status, as reflected by serum albumin, warrants careful interpretation. In the ACS‐NSQIP cohort, abnormal albumin levels were associated with increased odds of POD, consistent with prior evidence linking hypoalbuminemia to adverse surgical outcomes (Gibbs et al. 1999). In the TMUH cohort, however, only the category of missing albumin values was associated with lower odds of POD. This finding is unlikely to reflect a protective biological effect and more plausibly reflects nonrandom laboratory testing, whereby albumin measurements may have been preferentially ordered for patients perceived to be at higher clinical risk. Consequently, the albumin findings in the TMUH cohort should be interpreted cautiously.

COPD may reflect underlying systemic inflammation or reduced physiological reserve, both of which are relevant considerations in perioperative vulnerability to delirium (Cunningham and Hennessy 2015; Szylińska et al. 2020). In the present study, COPD and impaired renal function, as reflected by abnormal eGFR, were associated with POD in univariate analyses but did not retain independent associations in multivariable models in either cohort. This attenuation may reflect limited statistical power, cohort‐specific risk profiles, or overlapping predictive information captured by broader measures of physiological burden, such as ASA physical status classification. Interpretation of renal function deserves particular caution. eGFR was calculated using the Cockcroft–Gault formula, which incorporates age and body weight. Differences in age distribution and lower BMI in the TMUH cohort may therefore have influenced eGFR estimates, potentially introducing measurement artifacts rather than reflecting true differences in renal function across cohorts.

Preoperative WBC count may serve as a surrogate marker of immune activation or subclinical infection, mechanisms that have been implicated in delirium pathophysiology through neuroinflammatory pathways (Cunningham and Hennessy 2015). In our analyses, abnormal WBC count showed consistent associations with POD in univariate analyses across both datasets and remained independently associated with POD in the ACS‐NSQIP cohort. The absence of a similar association in the TMUH cohort may be attributable to limited sample size, reduced variability in laboratory values, or a lower burden of systemic inflammation in this population. These findings underscore the importance of interpreting regression results within the context of biological plausibility and cohort‐specific clinical characteristics. Across both cohorts, ASA physical status classification demonstrated a consistent association with POD, reinforcing its role as an integrative indicator of baseline health status and physiological resilience. ASA classification captures the cumulative impact of comorbid disease burden and functional reserve, factors that are central to perioperative risk stratification (Kang et al. 2020).

Differences in operative and baseline characteristics between cohorts further contextualize these findings. Patients in the ACS‐NSQIP cohort experienced longer operative times, potentially reflecting greater surgical complexity, whereas TMUH patients demonstrated higher preoperative albumin levels, suggestive of better nutritional status. Observed differences in platelet counts between cohorts may be related to demographic and ethnic variation rather than delirium‐specific mechanisms, as prior studies have documented population‐level differences in platelet distribution (Msaouel et al. 2014).

Several limitations should be acknowledged. First, delirium assessment was not universally applied to all surgical patients and was conducted as part of routine clinical workflows, limiting generalizability and precluding population‐based incidence estimation. Second, surgical procedures were heterogeneous, which may have diluted procedure‐specific risk signals. Third, laboratory variables were limited to those routinely available in both datasets, and missing laboratory data, particularly serum albumin in the TMUH cohort, introduced interpretive challenges despite efforts to preserve sample size.

Overall, these findings suggest that associations between clinical characteristics and POD may vary across healthcare systems and clinical contexts. While some factors, such as age and ASA classification, consistently reflect vulnerability, others appear to be context dependent. These results underscore the importance of cautious interpretation of regression findings and highlight the need for locally informed risk stratification approaches.

## 5 Conclusion

POD remains a common and clinically significant complication among older surgical patients. In this comparative analysis of clinically evaluated cohorts, we identified both shared and context‐specific associations with POD. These findings highlight the importance of considering local clinical practices and patient characteristics when interpreting delirium risk factors. Future prospective studies with standardized assessment protocols are warranted to validate these associations and to inform the development of context‐specific risk stratification and preventive strategies aimed at improving perioperative care for the oldest–old.

## Author Contributions

**Shan Hung**: literature review, interpretation of results, and drafting of the manuscript. **Chih‐Yau Chang**: acquistion an analysis of data, and interpretation of results. **Kuo‐Hsuan Chung**: literatur review, concetpiton and design of the study, supervision of the fieldwork, interpretation of results, and review of the manuscript.

## Funding

The authors have nothing to report.

## Ethics Statement

This study was conducted in accordance with the Declaration of Helsinki and was approved by the Institutional Review Board of Taipei Medical University (IRB No. N202308020, approved September 10, 2023). The requirement for informed consent was waived due to the retrospective nature of the study.

## Conflicts of Interest

The authors declare no conflicts of interest.

## Supporting information




**Supplementary Table S1**:brb371332‐sup‐0001‐TableS1.docx

## Data Availability

The datasets analyzed during the current study are available from the corresponding author upon reasonable request.
