# Supplementary material for: Postoperative Delirium in the Oldest–Old: Parallel Analyses of Institutional and National Surgical Cohorts
Source: Brain Behav. 2026 Apr 24;16(4):e71332. doi: 10.1002/brb3.71332 (PMC13109036; doi:10.1002/brb3.71332)
Supplement: Supplementary file 1 — Supplementary Table S1:brb371332‐sup‐0001‐TableS1.docx [file BRB3-16-e71332-s001.docx]

Supplementary Table S1. Comparison of demographic, clinical, and laboratory characteristics among patients with postoperative delirium in the ACS-NSQIP and TMUH cohorts.

|  |  | ACS-NSQIP  (n=147) | |  | TMUH  (n=65) | |  | *P* value |
| --- | --- | --- | --- | --- | --- | --- | --- | --- |
|  |  | n | % |  | n | % |  |  |
| Age | |  |  |  |  |  |  | <.001** |
|  | ≧90 | 0 | 0.00 |  | 13 | 20.00 |  |  |
|  | 85~90 | 28 | 19.05 |  | 11 | 16.92 |  |  |
|  | 80~85 | 48 | 32.65 |  | 17 | 25.15 |  |  |
|  | 75~80 | 71 | 48.30 |  | 24 | 36.92 |  |  |
| Abnormal eGFR | |  |  |  |  |  |  | 0.035* |
|  |  |  |  |  |  |  |  |  |
|  | No | 133 | 90.48 |  | 52 | 80.00 |  |  |
|  | Yes | 14 | 9.52 |  | 13 | 20.00 |  |  |
| Abnormal pre-operative serum albumin | | |  |  |  |  |  | <0.001** |
|  |  |  |  |  |  |  |  |  |
|  | No | 86 | 58.50 |  | 54 | 80.08 |  |  |
|  | Yes | 61 | 41.50 |  | 11 | 16.92 |  |  |
| ASA class | |  |  |  |  |  |  | 0.031† |
|  | I | 0 | 0 |  | 0 | 0 |  |  |
|  | II | 14 | 9.52 |  | 10 | 15.38 |  |  |
|  | III | 96 | 65.31 |  | 30 | 46.15 |  |  |
|  | IV | 35 | 23.81 |  | 25 | 38.46 |  |  |
|  | V | 2 | 1.36 |  | 0 | 0 |  |  |

|  | ACS-NSQIP | |  | TMUH | |  | *P* value |
| --- | --- | --- | --- | --- | --- | --- | --- |
|  | *M* | *SD* |  | *M* | *SD* |  |  |
| Age | 80.42 | 4.02 |  | 84.14 | 7.39 |  | <0.001** |
| BMI | 26.00 | 8.74 |  | 23.77 | 4.01 |  | 0.012* |
| Operating time | 193.3 | 127.8 |  | 130.3 | 138.7 |  | 0.002** |
| eGFR | 67.96 | 30.60 |  | 63.81 | 41.45 |  | 0.529 |
| Pre-operative serum albumin | 3.61 | 0.77 |  | 3.48 | 0.68 |  | 0.430 |
| Pre-operative WBC count | 8.94 | 5.07 |  | 8.41 | 4.5 |  | 0.537 |
| Pre-operative platelet count | 238.0 | 100.1 |  | 203.5 | 85.39 |  | 0.041* |

Footnotes:

This table is intended for descriptive comparison only and was not used for model development. Data are presented as mean ± standard deviation for continuous variables and number (%) for categorical variables. P-values were calculated using independent-samples t-test for continuous variables and chi-square test for categorical variables, unless otherwise specified. †Fisher’s exact test.

*Significance levels: p < 0.05; **p < 0.01.

Abbreviations:

ACS-NSQIP, American College of Surgeons National Surgical Quality Improvement Program; ASA, American Society of Anesthesiologists Physical Status Classification System; BMI, Body Mass Index; CHF, Congestive Heart Failure; COPD, Chronic Obstructive Pulmonary Disease; eGFR, Estimated Glomerular Filtration Rate; M, mean; n, number; SD, standard deviation; TMUH, Taipei Medical University Hospital; WBC, White Blood Cell (count).
